# Blunted cardiovascular responses in individuals with type 2 diabetes and hypertension during cold and heat exposure

**DOI:** 10.3389/fphys.2025.1558471

**Published:** 2025-04-28

**Authors:** Mojdeh Rafieian, Erlend Hoftun Farbu, Anje Christina Höper, Rasmus Valtonen, Henna Hyrkäs-Palmu, Juha Perkiömäki, Craig Crandall, Jouni J. K. Jaakkola, Tiina Maria Ikäheimo

**Affiliations:** ^1^ Department of Community Medicine, Faculty of Health Sciences, UiT the Arctic University of Norway, Tromsø, Norway; ^2^ Department of Occupational and Environmental Medicine, University Hospital of North Norway, Tromsø, Norway; ^3^ Research Unit of Biomedicine and Internal Medicine, University of Oulu, Oulu, Finland; ^4^ Medical Research Center Oulu, Oulu University Hospital and University of Oulu, Oulu, Finland; ^5^ Research Unit of Population Health, University of Oulu, Oulu, Finland; ^6^ Institute for Exercise and Environmental Medicine, Texas Health Presbyterian Hospital Dallas and University of Texas Southwestern Medical Center, Dallas, TX, United States; ^7^ Finnish Meteorological Institute, Helsinki, Finland

**Keywords:** type 2 diabetes, cold exposure, heat exposure, cardiovascular responses, blood pressure, heart rate, rate pressure product, hypertension

## Abstract

**Introduction:**

The effect of type 2 diabetes (T2D) on indices of cardiovascular function during exposure to cold or hot environmental temperatures is not well known. Therefore, the aim of our study was to assess the effect of short-term whole-body cold and heat exposure on the cardiovascular responses in individuals with T2D.

**Material and methods:**

10 participants with T2D and hypertension (mean age 64 ± 4 years) and 10 controls (mean age 63 ± 5 years) underwent 90 min of whole-body exposure to cold (10°C; 10% relative humidity) and heat (40°C; 50% relative humidity) in a randomized sequence on differing days. Central and brachial blood pressure (BP), heart rate (HR), and skin blood flow were measured before, during, and after the exposure.

**Results:**

During cold exposure, subjects with T2D exhibited a smaller increase in central (14 (CI 95%:3, 23) vs. 43 (CI 95%:32, 53) mmHg, p < 0.05) and brachial systolic BP (12 (CI 95%:1, 22)) vs. 40 (CI 95%:30, 51) mmHg, p < 0.05) compared to controls. The corresponding reduction in HR in the cold was also less in T2D compared to controls (5 (CI 95%: 10, 0.02) vs. 9 (CI 95%: 14, −4) bpm, p < 0.05). Heat exposure reduced central and brachial BP similarly in both groups. However, the heat-related increase in HR was less pronounced in T2D subjects compared to controls (7 (CI 95%:1, 13) vs. 14 (CI 95%: 9, 19) bpm, p < 0.05). Finally, the magnitude of the increase in skin blood flow was less in the heat in T2D subjects (+210 (CI 95%: 41, 461) vs. +605 (CI 95%: 353, 855) PU, p < 0.05).

**Discussion:**

T2D attenuated cardiovascular responses, such as BP and HR during short-term exposure to cold, and HR and skin blood flow during short-term exposure to heat. These observations suggest impaired capacity to respond to environmental temperature extremes in individuals with T2D.

## Introduction

Type 2 diabetes (T2D) is characterized by impaired insulin secretion and resistance (Roden and Shulman, 2019). This condition often leads to both microvascular and macrovascular complications, with cardiovascular disease being the predominant cause of mortality and morbidity (Mansour et al., 2023; De Grauw et al., 1995). Hypertension is co-occurring in at least half or even two-thirds of patients with T2D (Hajat et al., 2017; Al-Shihabi et al., 2023). The distinct features of T2D include beta-cell dysfunction and hyperglycemia, and in those of hypertension, vascular remodeling and pressure natriuresis impairments (Hajat et al., 2017; Al-Shihabi et al., 2023). However, these conditions share several pathophysiological responses, such as endothelial dysfunction, vascular inflammation, arterial changes, atherosclerosis, abnormal lipid levels, and obesity (Al-Shihabi et al., 2023). Both conditions also contribute to overlapping micro and macrovascular complications, which can together further magnify cardiovascular instability compared to each condition in isolation (Kenny et al., 2016). Their connection is primarily driven by common biological mechanisms, including overactivation of the renin-angiotensin-aldosterone system, oxidative stress, chronic inflammation, and immune responses (Al-Shihabi et al., 2023; Yardley et al., 2013). Therefore, assessing the combined impact of T2D and hypertension on cardiovascular responses, particularly under thermal stress, is important for understanding their full physiological burden.

Epidemiological studies suggest that individuals with T2D may be vulnerable to both low and high temperatures, as evidenced by increases in healthcare visits and mortality (Hajat et al., 2017; Al-Shihabi et al., 2023) during such conditions. Several processes, which are not yet fully understood, may account for the increased sensitivity to temperature extremes observed in individuals with T2D (Kenny et al., 2016; Yardley et al., 2013; Greaney et al., 2016; Cramer et al., 2022). These processes likely involve changes across multiple regulatory functions; i.e., the nervous system, evidenced by autonomic dysfunction, peripheral neuropathy; the cardiovascular system, characterized by reduced heart rate variability, endothelial dysfunction, arterial stiffness, and a tendency for increased blood clotting; and the metabolic system, indicated by insulin resistance and elevated blood glucose levels (Greaney et al., 2016; Spallone et al., 2011; Thorp and Schlaich, 2015). These alterations could impair the body’s ability to effectively regulate temperature under hot or cold conditions (Kenny et al., 2016).

Hypertension further complicates these regulatory impairments by disrupting autonomic function. Increased sympathetic nervous system activity in hypertension alters baroreflex mechanisms, reducing sensitivity and impairing heart rate modulation and vasomotor control (Greaney et al., 1985; Bairey et al., 2015; Narkiewicz and Grassi, 2008; Cat et al., 2001). Additionally, hypertensive individuals exhibit a diminished reflex reduction in muscle sympathetic nerve activity during blood pressure elevations, possibly due to arterial structural changes or neurohormonal factors (Greaney et al., 1985; Matsukawa et al., 1991). Furthermore, hypertension can lead to structural changes in the left ventricle, such as hypertrophy, as an adaptive response to increased pressure load, aiming to normalize myocardial wall stress. These changes can result in diastolic dysfunction and other cardiac complications (Mayet and Hughes, 2003) and may contribute to altered cardiovascular responses during cold and/or heat exposure.

The specific mechanisms responsible for alterations in cardiovascular responses to varying environmental temperature in individuals with T2D remain poorly understood (Kenny et al., 2016; Yardley et al., 2013; Greaney et al., 2016). Only a few controlled studies have examined whole-body responses to heat exposure, suggesting that persons with T2D may experience earlier heat strain (Wick et al., 1985). Furthermore, to our knowledge, no studies have addressed the effects of whole-body cold exposure on individuals with T2D. Given these significant gaps in knowledge, this study aimed to investigate the changes in key cardiovascular responses in individuals with combined T2D and hypertension during exposure to both cold and hot environments. Based on previous epidemiological and physiological studies (B38 et al., 2013; Laugesen et al., 2016; Carrillo et al., 2016), we expected blunted cardiovascular responses of persons with T2D when exposed to both conditions.

## Materials and methods

During the winter and spring of 2021, the laboratory study was conducted at the University of Oulu in Finland. The Ethics Committee of Oulu University Hospital District (EETTMK:199/2016) approved the study, and it was registered in the Clinical Trials database under the identifier NCT04698200. Additionally, the Regional Committee for Medical Research Ethics (REK) in Norway granted approval for the secondary data analysis of this investigation (Ref.nr. 2023/672049), and Norwegian Agency for Shared Services in Education and Research has assessed the form for processing personal data (Ref. No. 800577). Adherence to the ethical standards outlined in the Declaration of Helsinki was ensured, with participants providing written informed consent before involvement.

### Participants

In 2019, 957 men aged 40–79, living in Northern Ostrobothnia, were randomly selected for the study from the Finnish Population Register. Each was informed about the study through a mailed letter. Subsequently, successful telephone contacts were established with 663 of these individuals. A total of 304 did not meet the inclusion criteria, and 335 declined to participate upon contact. Of those contacted, 24 men were found eligible to participate in either the diabetes or control group. Additionally, two individuals were recruited through advertisements and email lists. Consequently, after the recruitment process, there were 13 individuals in the diabetes group and 13 in the control group. However, three participants from the diabetic group and three participants from the control group were subsequently excluded due to medication use, arrhythmias detected during the first measurement, or inability to be contacted after the initial measurement. The inclusion criteria for the diabetes group included a disease duration of at least 2 years, diagnosed hypertension, HbA1c levels from 7% to 10% (53–86 mmol/mol), and no history of smoking or active retinopathy. Thus, in this study, the term T2D refers to persons having both clinical conditions of T2D and hypertension. Persons without T2D, hypertension, and no history of smoking were asked to participate as controls. We aimed to match these groups as closely as possible regarding age and BMI. After screening for eligible participants, the study finally involved 10 people with T2D and 10 healthy control subjects.

### Assessment of baseline characteristics

All measurements were conducted at the same time of the day, starting in the morning. The subjects were advised to refrain from strenuous physical activity for 24 h, alcohol for 48 h, and coffee or caffeine-containing drinks for 2 h prior to the experiments. The participants were also instructed to eat breakfast as usual and take their medication(s). Upon arrival to the laboratory, each subject’s height and weight were recorded. Body fat percentage was estimated using a four-site skinfold measurement approach (triceps, biceps, subscapular, and suprailiac) (Durnin and Womersley, 1974). The hydration status was assessed by all participants by the PAL-10S refractometer (ATAGO, Tokyo, Japan) before the experimental exposures. None of the participants were dehydrated based on each having a urine specific gravity of less than 1.020. Blood glucose levels were checked with the Accu-Chek Aviva glucose meter (Roche Diabetes Care, Inc.) before and after the experiments with no indications of hypoglycemia. Finally, they completed a questionnaire inquiring about health and lifestyle-related factors as well as medications used. During both cold and heat trials, the participants were dressed lightly with shorts, socks, t-shirt and sandals with an insulation value of approximately 0.26 relative thermal insulation unit (ISO9920, 2007). Participants were equipped with eight thermistors to measure skin temperatures, 15 lead electrocardiogram (ECG) to continuously measure heart rate, and any possible ST elevation or depression, and a cuff to measure brachial blood pressure. Peripheral artery disease (PAD) status was assessed by ankle brachial index (ABI). The ABI was measured from both arms and ankles. The outcome was expressed as a ratio, with the ankle systolic pressure as the numerator and the brachial pressures as the denominator. Each leg’s ABI is determined independently, and the lower value of the two is used as the patient’s result. None of the participants had evidence of PAD based on the ankle brachial index. Experimental protocol. Thermal detection and pain thresholds were assessed using the Medoc Pathway model CHEPS system. Cold and heat detection thresholds were recorded separately from the thenar eminence (hand) and plantar surface (foot) by a thermode eliciting decreasing and increasing temperatures until the participant detect the sensation as heat or cold, with a reference temperature of 30°C, and a maximum exposure of 50°C for heat and 0°C for cold. Pain detection was assessed with the same equipment by increasing the heat intensity until the participant reported pain. The monofilament test was employed to assess possible neuropathy and loss of protective sensation. During the procedure, a 10-g filament was applied to three specific points on the foot to evaluate sensory response. The filament is pressed against the skin at a perpendicular angle until it bends, testing both feet in a consistent manner. A normal sensory response is indicated by detection at all testing points, while a lack of sensation at any point suggests neuropathic impairment.

**FIGURE 1:**
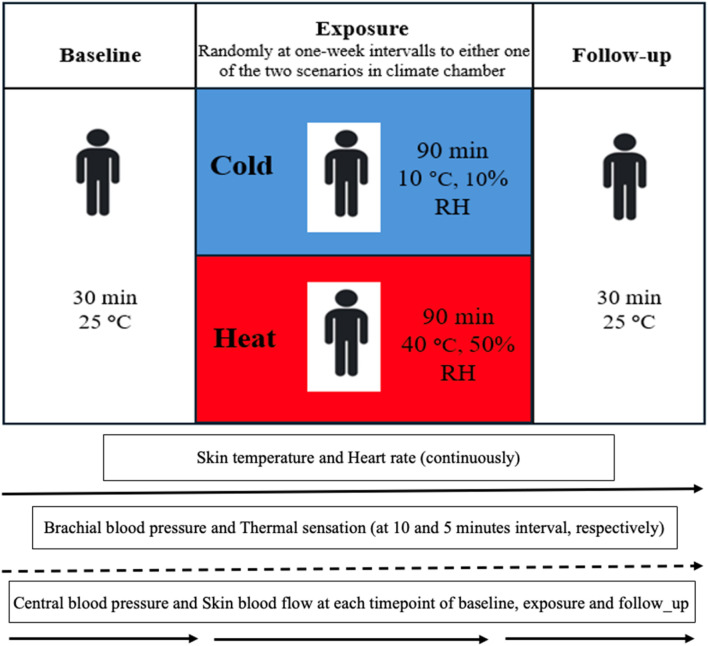
Experimental protocol diagram of three time points of baseline, exposure and follow-up and measured variables. Abbreviations; RH, relative humidity; °C; degrees Celsius.

**FIGURE 2:**
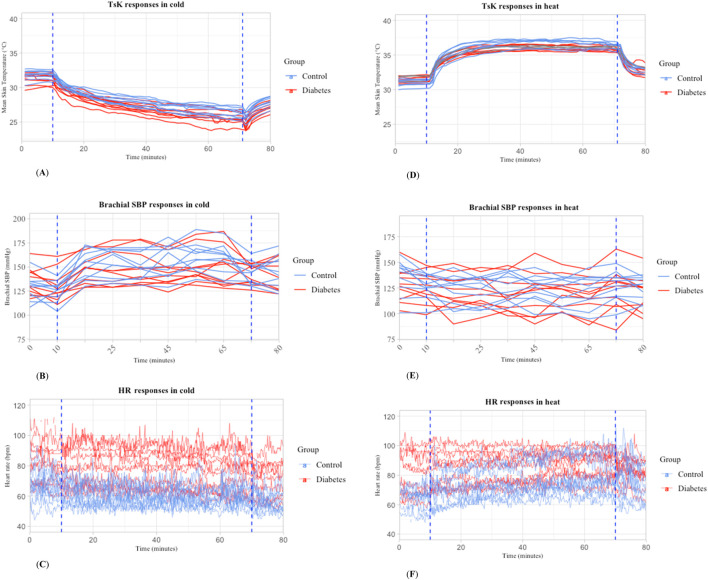
Mean skin temperature, heart rate, and brachial systolic blood pressure during whole-body exposure to cold **(A,B,C)** and heat **(D,E,F)** in individuals with T2D (n = 10) and controls (n = 10). Abbreviation: TsK: mean skin temperature; HR: heart rate; SBP: systolic blood pressure.

Regarding the baseline assessment, the subjects were seated for 30 min in a neutral environment (25°C). Following this period, they underwent exposures while seated to whole body cold (10°C; 10% relative humidity) or heat (40°C; 50% relative humidity) for 90 min each—in a randomized sequence with a 1-week interval between the two sessions. Subsequently, after cold/heat exposure they were again seated for 30 min in a neutral environment (25°C) as a follow-up (Figure 1). The exposures were designed to trigger thermogenesis in the cold and sweating in the heat, and both were expected to activate cardiovascular responses. Skin temperature and heart rate were continuously measured from baseline to follow-up. Brachial blood pressure and thermal sensations were measured at each time point at 10- and 5-min interval respectively. Central blood pressure and skin blood flow were measured for 5 min during baseline, after 60 min of exposure, and after 15 min of follow-up (Figure 1). Skin temperature, heart rate responses, and brachial systolic blood pressure were presented for each individual over 10 min of baseline, at 60 min of exposure, and the first 10 min of follow-up for both thermal conditions - see Figure 2.

### Skin temperature and thermal sensations

Skin temperature was measured by thermistors (NTC DC95, Digi-Key, Thief River Falls, MN, United States) attached at eight sites, with data being collected every 1 minute using temperature data loggers (SmartReaderPlus; Acr Systems Inc., BC, Canada). The mean skin temperature (Tsk) was derived using the formula: Tsk = [0.07 forehead +0.175 right scapula +0.175 left upper chest +0.07 right arm +0.07 left arm +0.05 left hand +0.19 right anterior thigh +0.2 left calf] (ISO 9886) (ISO9886, 2004). Thermal sensations were asked from the participants according to subjective judgment scales (ISO 10551) (ISO10551, 2019) once at baseline and follow-up, as well as at 5-minute- intervals throughout the exposures to heat or cold temperatures.

### Cardiovascular parameters

#### Central aortic blood pressure

The measurement of central hemodynamics was conducted using radial artery applanation tonometry. This involved placing a tonometry pressure sensor (SPC-301; Millar Instruments, Houston, TX, United States) directly on the radial artery of right arm. The sensor captured and digitally recorded the pulse from the radial artery. The central aortic blood pressure was calculated from the pressure curve of the radial artery, using the mean of three systolic and diastolic brachial blood pressure readings for calibration. This calculation was performed through a mathematical algorithm approved by the US Food and Drug Administration (SphygmoCor Px; AtCor Medical, Sydney, Australia). Data quality was maintained by discarding any measurements where the built-in quality control operator index (SphygmoCor Px) fell below 75%, ensuring minimal variation in pulse height, diastolic pressure, and the pressure wave shape during systole.

#### Wave reflection parameters

The pulse pressure (PP) is the difference between the systolic and diastolic blood pressure (Tang et al., 2020). The augmentation index (AI%) represents the proportional contribution of reflected waves to systolic pressure, calculated as the augmented pressure (AP) divided by pulse pressure (PP), expressed as a percentage. AI75 is AI adjusted to a standardized heart rate of 75 beats per minute (bpm) to account for heart rate dependency. P1 corresponds to the pressure at the first systolic shoulder of the aortic waveform, marking the peak of the incident pressure wave, while P2 reflects the second systolic peak caused by wave reflection. AP is defined as the difference between P2 and P1 (P2 − P1) (Laurent et al., 2006). Ejection duration (ED) measures the time in the cardiac cycle when the left ventricle contracts to push blood through the aortic valve into circulation (Tocci et al., 2021). Reflected wave transit time (Tr) is the time from the initial pressure upstroke to the inflection point, indicating the round-trip travel time of the reflected wave (Afkhami and Johnson, 2021).

#### Brachial blood pressure

Brachial blood pressure was recorded using (Schiller BP 200 Plus; Schiller, Baar, Switzerland) from the left arm. For the measurement, the subjects sat with their arms supported and positioned at heart level.

#### Electrocardiagram

ECG was continuously recorded and monitored using a 15-lead system (Cardiosoft V6.71, GE Healthcare, Freiburg, Germany). Electrode placement for the ECG adhered to the standard 12-lead configuration along with X, Y, Z leads. Analysis of the signals was conducted using specialized software created in Matlab (MathWorks, Natick, MA). Abnormal and ectopic beats were omitted from the analysis.

#### Rate pressure product and subendocardial viability ratio

The central and brachial rate-pressure products (aRPP and RPP, respectively), which provides an index of the heart’s workload, were calculated by multiplying the heart rate by the respective systolic blood pressures (Zhou et al., 2024). The subendocardial viability ratio (SEVR%) measured as the ratio of the time-integrated central aortic blood pressure during systolic and diastolic phases, reflecting the relationship between myocardial oxygen supply and demand.

#### Skin blood flow

Skin blood flow was measured from glabrous skin of the finger using laser Doppler flowmetry (LDF; OxyFlo ProXL). The sensor was placed on finger and kept in place throughout baseline, exposure, and follow-up. The sensor’s location was marked on the skin with a pen to facilitate returning the probe to the same location for the subsequent cold or heat exposures.

The LDF signal pre-processing in Matlab included noise removal with a 2.5 Hz IIR Butterworth filter, baseline correction *via* polynomial detrending, and signal normalization. Outliers were replaced using a 2.25 SD threshold, and signals were downsampled to 10 Hz. Frequency-domain analysis used Continuous Wavelet Transform (CWT) with the Analytic Morlet Wavelet (amor) in the 0.0095–2 Hz range.

#### Statistical analysis

We used the Student’s t-test for continuous variables and the chi-square test or Fisher’s exact test for nominal variables to compare means between the T2D and the control group related to their baseline characteristics. Within each thermal condition, a mixed-effects model, using the “lmer” package, was utilized to analyze cardiovascular responses (central and brachial BP, HR, aRPP, RPP, and SEVR). The analysis looked at within-individual effects by examining how each person’s responses changed over time (from baseline to cold/warm exposure and follow-up). It also examined between-group effects by comparing the cardiovascular responses of the T2D and control groups at each timepoint. In a subsequent analysis, body mass index (BMI) was added as a covariate to further explore its impact (Supplemental material, Tables 1, 2). The results are presented as means or estimated marginal means, along with their standard deviations (SD) or 95% confidence intervals. Categorical variables were described through their relative frequencies. Statistical significance was set at p < 0.05. All statistical analyses were conducted using R release (2022.07.1) (R, 2022).

**TABLE 1:** Participants characteristics of people with T2D and controls.

Variable	T2D subjects (n = 10)	Control subjects (n = 10)	P Values
Age, y	64 ± 4.14	63 ± 5.42	>0.05
Height, cm	176.2 ± 5.7	176.3 ± 7.1	>0.05
Weight, kg	93.6 ± 16.3	79 ± 8.19	<0.05
BMI, kg/m^2^	30.1 ± 4.8	25.4 ± 2.1	<0.05
Fat percentage (%)	31.6 ± 6.5	25.2 ± 3.7	<0.05
Health perception			<0.05
Very good	12.5% (n = 1)	30% (n = 3)	
Fairly good	25% (n = 2)	60% (n = 6)	
Average	50% (n = 4)	10% (n = 1)	
Somewhat poor	12.5% (n = 1)	-	
Alcohol consumption %			>0.05
At least once a month	75% (n = 6)	60% (n = 6)	
Less than once a month	25% (n = 2)	10% (n = 1)	
Stopped drinking	-	20% (n = 2)	
Never used alcohol	-	10% (n = 1)	
Physical activity during leisure time			>0.05
Physical activity like running, at least 4 hours per week	25% (n = 2)	50% (n = 5)	
Physical activity like running, average 3 hours per week	37.5% (n = 3)	50% (n = 5)	
No physical activity	37.5% (n = 3)	-	
Physical activity at work			>0.05
Working involves mainly sitting	85.7% (n = 6)	55.6% (n = 5)	
Walking or caring heavy items	-	33.3% (n = 3)	
Walking, lifting, and caring heavy items and climbing stairs	14.3% (n = 1)	11.1% (n = 1)	
Blood pressure medications^a^			
Calcium blocker	54.5% (n = 6)		
Beta blocker	9.1% (n = 1)		
Angiotensin II receptor blockers	18.2% (n = 2)		
Angiotensin-converting-enzyme inhibitors	18.2% (n = 2)		
Diabetes medications			
Biguanides	50% (n = 7)		
SGLT2 Inhibitors	21.5% (n = 3)		
GLP-1 Receptor Agonists	7.1% (n = 1)		
DPP-4 Inhibitors	14.3% (n = 2)		
Insulin & Insulin Analogues	7.1% (n = 1)		
ABI	1.14 ± 0.05	1.18 ± 0.05	>0.05
Cold, warm and pain detection thresholds (hand)			
CDT	26.3 ± 4.47	28.8 ± 0.360	<0.05
CP	9.76 ± 6.89	9.92 ± 7.40	>0.05
WDT	35.7 ± 4.96	32.4 ± 0.700	<0.05
HP	45.9 ± 3.28	45.5 ± 3.12	>0.05
Cold, warm and pain detection thresholds (foot)			
CDT	17.9 ± 10.3	24.7 ± 4.06	<0.05
CP	3.21 ± 4.91	5.59 ± 6.17	>0.05
WDT	43.3 ± 4.87	41.4 ± 2.41	>0.05
HP	47.8 ± 1.89	47.7 ± 1.72	>0.05
Monofilament Test			
Number of individuals with at least one problematic point in vibration perception	6	7	>0.05

Continuous variables are presented as mean values and their SDs. categorical variables are described by relative frequencies.

Abbreviations: BF% body fat percentage; BMI, body mass index; ABI, ankle brachial index test; CDT, cold detection threshold; CP, cold pain threshold; WDT, warm detection threshold; HP, heat pain threshold.

^a^ Missing information for T2D: medication; n = 3.

**TABLE 2:** Blood pressure, heart rate and finger skin blood flow response in subjects with T2D and controls in response to whole-body exposure to cold (10°C) at rest: The values represent Estimated Marginal Means (95% Confidence Interval) Calculated by Mixed-Effect Models.

	T2D			Control			P-value for T2D vs. control		
Variable	Baseline N = 10	Cold exposure N = 8	Follow-up N = 10	Baseline N = 10	Cold exposure N = 8	Follow-up N = 9	Baseline	Cold exposure	Follow_up
Central aortic SBP, mmHg	115 (105–125)^b^	129 (118–139)	124 (113–134)	112 (102–122)^b^ ^,^ ^c^ ^,^ ^d^	155 (145–165)	125 (115–135)	0.65	0.00	0.87
Central aortic DBP, mmHg	80.1 (73.6–86.5)	85.3 (78.6–92.1)	81.4 (74.6–88.2)	77.9 (71.4–84.3)^b^ ^,^ ^d^	92.3 (85.6–99.1)	76.5 (69.9–83.1)	0.62	0.15	0.30
Brachial SBP, mmHg*	130 (120–141)^b^	142 (131–153)	135 (124–146)	123 (112–133)^b^ ^,^ ^c^ ^,^ ^d^	163 (152–174)	133 (122–144)	0.31	0.01	0.74
Brachial DBP, mmHg^*^	79.1 (72.8–85.4)	84.2 (77.6–90.9)	80.2 (73.5–86.9)	76.9 (70.5–83.2)^b^ ^,^ ^d^	91.8 (85.1–98.4)	75.8 (69.3–82.3)	0.62	0.11	0.35
Central aortic PP, mmHg	35.2 (27.6–42.9)	43.5 (35.5–51.5)	42.5 (34.5–50.6)	34.5 (26.9–42.1)^b^ ^,^ ^c^ ^,^ ^d^	62.7 (54.7–70.7)	48.6 (40.8–56.4)	0.89	0.00	0.28
Brachial PP, mmHg	51 (42.3–59.6)	57.7 (48.7–66.7)	55.2 (46.2–64.3)	45.8 (37.2–54.5)^b^ ^,^ ^c^ ^,^ ^d^	71.3 (62.3–80.3)	57.2 (48.4–66)	0.3988	0.04	0.75
AP, mmHg	5.7 (2.14–9.3)^c^	10.5 (6.65–14.4)	11.25 (7.4–15.1)	8.09 (4.51–11.7)^b^ ^,^ ^c^ ^,^ ^d^	25 (21.18–28.9)	16.7 (12.99–20.4)	0.35	<0.0001	0.04
AI75%	18.1 (13.9–22.2)	21.9 (17.4–26.4)	21.6 (17.1–26.1)	18.4 (14.2–22.5)^b^ ^,^ ^c^	30.3 (25.8–34.7)	25.4 (21.1–29.7)	0.91	0.01	0.23
AI%	15.1 (9.9–20.3)^c^	21.6 (16–27.3)	23.5 (17.9–29.2)	24 (18.76–29.2)^b^ ^,^ ^c^	39.8 (34.22–45.5)	33.7 (28.3–39.1)	0.02	<0.0001	0.01
P1, mmHg	109 (101.6–117)^b^	118 (109.7–126)	112 (104.3–121)	104 (96.1–112)^b^ ^,^ ^d^	131 (122.4–139)	108 (99.8–116)	0.32	0.03	0.42
P2, mmHg	115 (105–125)^b^	129 (119–139)	124 (113–134)	112 (102–122)^b^ ^,^ ^c^ ^,^ ^d^	155 (145–165)	125 (115–135)	0.66	0.00	0.87
ED, ms	272 (258–286)^c^	283 (268–298)	292 (277–307)	289 (275–303)^b^ ^,^ ^c^	326 (311–341)	318 (303–332)	0.09	0.00	0.02
Tr, ms	79.8 (67–92.6)^b^ ^,^ ^c^	100.6 (87–114.3)	105.9 (92.2–119.6)	104.7 (91.9–117.4)^b^ ^,^ ^c^	141 (127.4–154.7)	134.9 (121.7–148.1)	0.01	0.00	0.00
HR,bpm	80.4 (74.5–86.4)^c^	75.2 (69.1–81.4)	70.8 (64.6–77)	63.9 (58–69.9)ab	55 (48.8–61.2)	57.4 (51.3–63.4)	0.00	0.00	0.00
aRPP,bpm xmm Hg	9220 (8405–10034)^d^	9509 (8667–10352)	8623 (7778–9468)	7188 (6374–8003)^b^ ^,^ ^d^	8552 (7712–9393)	7154 (6326–7982)	0.00	0.11	0.02
RPP, bpm xmm Hg	10414 (9460–11368)^c^ ^,^ ^d^	10507 (9518–11496)	9480 (8488–10472)	7871 (6917–8825)^b^ ^,^ ^d^	9000 (8013–9986)	7637 (6666–8608)	0.00	0.03	0.01
SEVR%	153 (141–164))^c^	157 (144–169)	165 (153–177)	197 (186–209))^c^	190 (177–202)	187 (175–199)	0.00	0.00	0.01
Skin blood flow, PU	384.9 (266.9–503)^b^ ^,^ ^c^	19.7 (−135.2–175) #	50.5 (−67.5–168)	578.3 (460.4–696)^b^ ^,^ ^c^	23.7 (−101.1–149)	56.3 (−61.6–174)	0.02	0.97	0.94
Cutaneous vascular resistance	0.31 (−5.27–5.90)	5.50 (−0.99–11.99)	2.83 (−3.15–8.82)	0.26 (−4.73–5.25)^b^	9.79 (4.52–15.07)	3.47 (−1.52–8.46)	0.99	0.31	0.87

Abbreviation: SBP, systolic blood pressure; DBP, diastolic blood pressure; PP, pulse pressure; AP, central augmented pressure; AI, unadjusted augmentation index; AI75, augmentation index adjusted to heart rate of 75 beats/minute; P1, first systolic pressure peak; P2, second systolic pressure peak; ED, ejection duration; Tr, reflection time; aRPP, central aortic rate–pressure product (heart rate × aortic systolic blood pressure); RPP, brachial rate–pressure product (heart rate × brachial systolic blood pressure); SEVR, subendocardial viability ratio.

^a^ the number listed for brachial SBP, under the “Cold Exposure” column corresponds to measurements taken 60 min after the cold exposure.

^b^ P < 0.05 baseline vs. exposure within that group.

^c^ P < 0.05 baseline vs. follow-up within that group.

^d^ P < 0.05 exposure vs. follow-up within that group.

# Missing information for Skin blood flow T2D: n = 4.

## Results

### Participants characteristics

Participants with T2D had in general higher weight, BMI and fat percentage, compared to controls. Individuals with T2D were more likely to rate their health as average, while those in the control group more often described their health as fairly good. Most people with type 2 diabetes in the study were taking calcium blockers (54.5%) to manage their blood pressure and biguanides (50%) to control their diabetes. There were no indications of peripheral artery disease (ABI-measurement) or neuropathy (monofilament test) among the individuals with T2D. Furthermore, thermal sensitivity testing showed increased cold sensitivity (hand, feet) and decreased heat sensitivity (hand), but no effects on heat or cold pain detection among them (Table 1).

### Exposure to cold

#### Thermal responses

After 1 hour of cold exposure, the mean skin temperature decreased more in subjects with T2D than in controls (approximately 5.8°C (CI 95%: 5,6) vs. 5.4°C (CI 95%:5,6), respectively) (p < 0.05). This variable also remained 0.8°C lower in T2D than the controls after 10 min of follow-up (p < 0.05) (Individual responses are shown in Figure 2A, and means of each group with 95% CI in Figure 1 supplementary material). Aligned with this finding, reported sensations of cold, were initially the same across both groups during the baseline and the cold exposure, but sensations of slight coolness were more dominant in T2D subjects during the follow-up.

#### Cardiovascular responses

As shown in Table 2, there was a smaller increase in central systolic blood pressure (SBP) following cold exposure in subjects with T2D compared to the controls (change from baseline to exposure was 14 (CI 95%:3,23) vs. 43 (CI 95%:32,53) mmHg) (p < 0.05). Brachial SBP mirrored the trends observed in central aortic systolic pressures during cold exposure, with an increase of 12 mmHg (CI 95%:1,22) in the T2D and by 40 mmHg (CI 95%:30,51) in the control group (p < 0.05) (Individual responses are shown in Figure 2B, and means of each group with 95% CI in Supplementary Figure S2). Heart rate demonstrated a lesser decrease during cold exposure in T2D compared with control subjects (5 (CI 95%: 10,0.02) vs. 9 (CI 95%: 14, −4) bpm). It should be noted that despite these changes, heart rate was significantly higher in subjects with T2D than in the controls at all three measurement points (p < 0.05) (Individual responses are shown in Figure 2C, and means of each group with 95% CI in Supplementary Figure S3).

Both central aortic and brachial pulse pressures (PP) increased in response to cold, but the increase was smaller in individuals with T2D compared to controls. Central PP was (8 (CI 95%:1,15) in T2D vs. 28 (CI 95%:21,35) mmHg) in control and brachial PP was (7 (CI 95%: 1,14) in T2D vs. 25 (CI 95%:18,33) mmHg) in control (p < 0.05). Central augmented pressure (AP) and the augmentation index (AI) also increased less in individuals with T2D compared to controls (5 (CI 95%:0.6,9) vs. 17 (CI 95%:13,21) mmHg) and (6% (CI 95%:0.4,13) vs. 16% (CI 95%:10,22)) respectively (p < 0.05). The first (P1) and second (P2) systolic pressure peaks showed a smaller increase in T2D patients than in controls during cold exposure (9 (CI 95%:2,15) vs. 27 (CI 95%:20,33) mmHg) and (14 (CI 95%:4,23) vs. 43 (CI 95%:34,52) mmHg) respectively (p < 0.05). Ejection duration (ED) and reflection time (Tr) also had a smaller increase in individuals with T2D compared to controls (11 (CI 95%: 3,24) vs. 37(CI 95%:23,50) ms) and (21 (CI 95%:7,35) vs. 36 (CI 95%:22,50) ms) respectively (p < 0.05).

Cold exposure resulted in a lesser increase in both aRPP and RPP in the T2D group compared to controls (aRPP: 289 (CI 95%: 450, 1029) vs. 1364 (CI 95%:629,2099) bpm × mmHg; RPP: 93 (CI 95%: 804, 991) vs. 1129 (CI 95%:236,2021) bpm × mmHg). During the follow-up phase, both aRPP and RPP decreased less in the T2D group compared to controls (aRPP: 886 (CI 95%: 1710,-63) vs. 1398 (CI 95%: 2209,-587) bpm × mmHg; RPP: 1027 (CI 95%: 2027,-28) vs. 1363 (CI 95%: 2347,-379) bpm × mmHg). SEVR was lower in individuals with T2D compared to the control group at all time points (p < 0.05) (Bar graphs showing the delta of cold exposure *versus* baseline for central SBP, aRPP, and SEVR can be found in the Supplementary Figures S4–6). No changes were observed in the ECG regarding ST elevation or depression at any time points in individuals from either the T2D or control group. Cold exposure decreased skin blood flow to a lesser extent among subjects with T2D compared with the control subjects (365 (CI 95%: 588,-143) vs. 554 (CI 95%: 752, −358) PU) (p > 0.05). However, there is some uncertainty about the true effect due to the lower baseline blood flow in T2D compared to the control group. As a result of cold exposure, T2D subjects showed a lesser increase in cutaneous vascular resistance compared to controls (+5.19 (CI 95%: 4.19,14.6) vs. +9.53 (CI 95%: 1.60,17.5)) (Bar graphs showing the delta of cold exposure *versus* baseline for skin blood flow and cutaneious vascular resistance can be found in the Supplementary Figures S7, 8).

### Exposure to heat

#### Thermal responses

After 1 hour of heat exposure, the mean skin temperature increased less in subjects with T2D than in controls (approximately 3.8°C (CI 95%: 4, −3) vs. 4.5°C (CI 95%: 5, −4), respectively) (p < 0.05) (Individual responses are shown in Figure 2D, and means of each group with 95% CI in Supplementary Figure S1). Warm sensations were initially the same across both groups in baseline and heat exposure, but T2D subjects reported being slightly warmer during the follow-up.

#### Cardiovascular responses

Heat exposure reduced central and brachial blood pressure to a similar extent in T2D and control subjects (Individual responses are shown in Figure 2E, and means of each group with 95% CI in Supplementary Figure S2). Similarly, none of the other parameters of central blood pressure differed between T2D and controls. The increase in heart rate was less pronounced in T2D subjects compared to controls (7 (CI 95%:1,13) vs. 14 (CI 95%: 9,19) bpm) (p < 0.05). Nonetheless, at all three measured timepoints, individuals with T2D exhibited higher heart rates than the controls (p < 0.05) (Individual responses are shown in Figure 2F, and means of each group with 95% CI in Supplementary Figure S3). Furthermore, the rise in both RPP and aRPP due to heat exposure was less significant in the T2D group than in the controls (RPP: 611 (CI 95%: 353,1574) vs. 1646 (CI 95%:830, 2462) bpm × mmHg; aRPP: 467 (CI 95%: 258,1192) vs. 1229 (CI 95%:615,1842) bpm × mmHg). Generally, the level of SEVR% was lower in T2D subjects than in controls at all three measurement points (p < 0.05). However, the reduction in SEVR% due to heat exposure was less in individuals with T2D compared to control subjects (reduction 8% (CI 95%: 22, 6) vs. 29% (CI 95%: 41,-16)) (p < 0.05) (Bar graphs showing the delta of exposure and baseline for central SBP, aRPP, and SEVR can be found in the Supplementary Figures S4–6).

No changes were observed in the ECG regarding ST elevation or depression at any timepoints in individuals from either the T2D or control group. T2D subjects showed a lesser increase in skin blood flow compared to controls in heat exposure (+210 (CI 95%: 41, 461) vs. +605 (CI 95%: 353, 855) PU). Similarly, during the follow-up, T2D subjects showed a lesser recovery of skin blood flow compared with controls (reduction 98 (CI 95%: 357, 163) vs. 279(CI 95%: 529,-27) PU). As a result of exposure to heat, T2D subjects showed a lesser decrease in cutaneous vascular resistance compared to controls (−0.02 (CI 95%: 0.27,0.23) vs. −0.37 (CI 95%: 0.62,-0.13)). Similarly, during the follow-up, T2D subjects showed a lesser recovery in cutaneous vascular resistance compared with controls (−0.04 (CI 95%: 0.30, 0.22) vs. −0.08 (CI 95%: 0.17, 0.34)). Notably, pre-heat stress baseline skin blood flows were lower among T2D subjects compared to controls. (Bar graphs showing the delta of heat exposure *versus* baseline for skin blood flow and cutaneious vascular resistance can be found in the Supplementary Figures S7, 8).

## Discussion

To our knowledge, this study is the first to report detailed cardiovascular responses in T2D under both cold and heat conditions of the same individuals. Our results suggest a dampened increase in blood pressure and reduction in heart rate during cold exposure in individuals with T2D compared to controls. Additionally, exposure to heat among the same individuals with T2D demonstrated a lesser increase in heart rate compared to the controls.

### Cardiovascular response to cold exposure

The cold exposure caused significant superficial cooling, as judged by decreased skin temperature of >5°C. Furthermore, skin temperatures decreased more in participants with T2D compared to controls and remained lower during the recovery period.

In both groups, we observed increased central aortic and brachial BP in cold exposure likely related to cold-induced vasoconstriction (Castellani and Young, 2016; Wilson, 2017). However, this increase in peripheral vascular resistance to cooling was lesser among persons with T2D compared with controls. Concerning blood pressures, those with T2D exhibited significantly lower responses to cold in several key parameters. That is, both central aortic and brachial pulse pressures increased in response to cold, but the magnitude of that increase was attenuated in individuals with T2D. Central augmented pressure and augmentation index, indicators of arterial wave reflection, were lower in individuals with T2D in response to cold exposure, suggesting altered vascular stiffness or wave reflection patterns. First and second systolic pressure peaks were lower among T2D than controls during cold exposure, indicating a blunted systolic pressure response. Ejection duration and reflection time relate to left ventricular systolic function and arterial wave reflection were also significantly lower in the T2D than control subjects. In summary, these findings indicate that individuals with T2D exhibit a diminished central blood pressure response to cold exposure compared to healthy controls. The lesser increase in BP observed among those with T2D could be due to reduced systemic vasoconstriction. This hypothesis is supported by the study of Stansberry et al. (1993) who examined the influence of local cooling of the hands on peripheral blood flow and showed reduced vasoconstriction among persons with T2D (Stansberry et al., 1997). We also observed a lesser cold-induced reduction in HR (bradycardia) among persons with T2D than controls. Bradycardia is commonly observed in studies employing whole-body cold exposure among healthy persons (Korhonen, 2006) and those with cardiovascular diseases (Hintsala et al., 2014; Valtonen et al., 2022). The underlying mechanism could be facial exposure to cold, which activates the trigeminal nerve, eliciting a vagal response and related bradycardia (Ikäheimo, 2018). Therefore, altered parasympathetic activity of persons with T2D could explain the dampened reduction in HR when exposed to cold. In addition, impaired overall ANS function, and, for example, reduced baroreflex sensitivity (La Rovere et al., 2008), could further contribute to the dampened HR response. However, these mechanisms need to be further studied. Consistent with our findings, reduced blood pressure and heart rate responses were also observed in streptozotocin-diabetic rats during whole-body cooling (Kilgour and Williams, 1998). It is important to note that HR remained higher in persons with T2D compared with controls throughout the baseline, cold exposure, and follow-up. This could relate to various factors, such as autonomic neuropathy, poor glycemic control, or physical deconditioning related to the disease (Balcıoğlu and Müderrisoğlu, 2015).

Our study detected increased RPP in the cold, which indicates increased cardiac strain (i.e., elevated myocardial workload and oxygen consumption) and relates primarily to the observed higher blood pressure (Segan et al., 2013; Castellani and Young, 2016; White, 1999). However, we demonstrated a lesser increase in RPP (both brachial and central) in individuals with T2D compared to controls, likely due to less of an increase in systolic BP among individuals with T2D. Consistent with our findings, Segan et al. (Segan et al., 2013) observed a diminished RPP response in individuals with T2D and cardiac autonomic neuropathy during a cold pressor test with local cooling of hands. However, it should be noted that the cold pressor test involves a strong sympathetic response and a painful stimulus that differs in this respect from the whole-body cold exposure used in our study. The observed smaller increase in RPP to whole-body cooling in those with T2D could be due to altered autonomic function or vascular reasons (e.g., endothelial dysfunction) but the reasons remain to be further investigated (Segan et al., 2013). Of note, a higher RPP was observed in T2D throughout the baseline, cold exposure, and follow-up likely suggesting autonomic dysfunction in the diabetic population (Foo et al., 2004). Another finding in our study was that SEVR was lower in T2D than controls at all time points, indicating a reduced myocardial oxygen supply/demand ratio, which is often associated with cardiovascular disease (Laugesen et al., 2016).

Finger skin blood flow decreased in all participants in response to cold, indicative of vasoconstriction (Stansberry et al., 1997). However, the magnitude of this reduction in cold was less in persons with T2D compared with controls (365 vs. 554 PU). Consistent with this finding, cutaneous vascular resistance also increased less in the cold among those with T2D. That said, it should be noted that finger skin blood flow values at baseline were lower in persons with T2D compared to controls. Hence, the seemingly blunted response to cold observed among T2D could relate to reaching minimal detectable values (basement effect) of blood flow earlier. Overall, reduced cutaneous blood flow in persons with T2D can be related to various factors, such as endothelial dysfunction, increased vascular tone, microvascular changes, and peripheral neuropathy (Petrofsky, 2011; Tikhonova et al., 2022; Vinik et al., 1992).

In summary, the dampened cardiovascular responses observed in individuals with T2D during cold exposure in our study may be attributed to altered autonomic regulation. This effect could depend on disease progression as reflex cutaneous sympathetic and vascular responses to rapid whole-body cooling are preserved in relatively healthy individuals with T2D (Strom et al., 2011). Altered autonomic nervous function can impair the typical sympathetic response to cold that may lead to attenuated vasoconstriction responses resulting in a lesser increase in BP and aortic RPP and brachial RPP compared to healthy individuals (Balcıoğlu and Müderrisoğlu, 2015; Rasmussen et al., 2022; McAuley et al., 2000). From a thermoregulatory perspective, blunted cardiovascular response in the cold could indicate a lesser possibility of those with T2D to respond to a lowering of environmental temperature. Eventually, this could lead to increased heat loss in cold environments.

### Cardiovascular response to heat exposure

The employed heat exposure at rest significantly increased mean skin temperature (approximately 4°C) which was lesser in T2D compared with controls (Figure 2D).

Exposure to heat similarly reduced brachial and central BP among all participants, indicating cutaneous vasodilation and reduced peripheral vascular resistance (Cheng and MacDonald, 1985). Concerning central aortic BP, none of the parameters differed between T2D and controls Table 3. This finding deviates from that observed from cold exposure which showed consistently blunted BP responses related to having T2D. HR increased in all participants, likely related to maintaining cardiac output resulting from cutaneous vasodilation (Cheng and MacDonald, 1985). However, the increase in HR was lesser among persons with T2D than the control group. Supporting the reduced HR response, we also observed a lesser increase in finger skin blood flow in response to heat among persons with T2D. This response could suggest a reduced ability for the superficial vessels to vasodilate in response to heat. Our results align with studies employing local heating or vasoactive agents, which showed lower maximal cutaneous vasodilatory and skin blood flow responses in persons with diabetes (Wick et al., 1985; Fujii et al., 2018; Veves et al., 1998; Wilson et al., 1992). Such a response could indicate impaired cardiovascular regulation and the ability to cope with heightened cardiac demands when exposed to heat (Kenny et al., 2016). Our findings differ from a previous study where HR responses were similar between T2D and controls in response to prolonged (3 h) passive heat in 44°C (Poirier et al., 2020). Of note, HR was higher throughout the baseline, exposure, and follow-up, which could be due to several reasons, such as impaired autonomic regulation or vascular dysfunction related to the disease. This might compromise the ability to properly adjust heart rate and vascular tone in response to heat stress, potentially related to the disease (Carrillo et al., 2016). Heat exposure increased both central and brachial RPP in all participants, but the overall increase was less in persons with T2D. This response is related to the dampened increase in HR observed in T2D. Both central and brachial RPP was higher in T2D in all time points, and where a reduced RPP response could also indicate lower myocardial workload and oxygen demand, which may be cardioprotective.

**TABLE 3:** Blood pressure, heart rate and finger skin blood flow response in subjects with T2D and controls in response to whole-body exposure to heat (40°C) at rest: The values represent Estimated Marginal Means (95% Confidence Interval) Calculated by Mixed-Effect Models.

	T2D			Control			P-value for T2D vs. control		
Variable	Baseline N = 8	Heat exposure N = 8	Follow-up N = 8	Baseline N = 10	Heat exposure N = 10	Follow-up N = 10	Baseline	Heat exposure	Follow_up
Central aortic SBP, mmHg	110 (102.6–118)	105 (97.4–113)	106 (98.2–114)	116 (108.9–124)	112 (104.2–119)	113 (105.8–121)	0.26	0.22	0.16
Central aortic DBP, mmHg	75.3 (69.0–81.5)	73.1 (66.9–79.3)	72.6 (66.2–78.9)	82.1 (76.3–87.9)^b^ ^,^ ^d^	78.4 (72.6–84.2)	81.9 (76.1–87.7)	0.11	0.21	0.03
Brachial SBP, mmHg^*^	128 (115–138)^b^ ^,^ ^c^	117 (107–127)	119 (109–128)	127 (118–137)	123 (113–132)	125 (116–135)	0.88	0.53	0.41
Brachial DBP, mmHg^*^	74.3 (68.1–80.5)	71.8 (65.6–78.1)	71.6 (65.3–77.9)	81.3 (75.5–87.1)	77 (71.2–82.8)	80.9 (75.1–86.7)	0.10	0.22	0.03
Central aortic PP, mmHg	34.7 (28.9–40.4)	31.7 (26.0–37.4)	32.8 (26.9–38.8)	34.2 (28.9–39.5)	33.2 (27.9–38.5)	31.4 (26.1–36.7)	0.90	0.70	0.71
Brachial PP, mmHg	51.9 (43.1–60.7)	49.6 (40.8–58.4)	48.7 (39.7–57.7)	45.90 (37.8–54.0)	48.8 (40.7–56.9)	45.2 (37.1–53.3)	0.31	0.89	0.56
AP, mmHg	4.6 (2.261–6.94)	3.14 (0.798–5.48)	3.8 (1.397–6.29)	7.70 (5.568–9.83)^b^	4.8 (2.668–6.93)	5.60 (3.468–7.73)	0.05	0.29	0.28
AI75%	13.9 (8.73–19.1)	15.1 (9.96–20.3)	14.3 (8.92–19.7)	15.8 (11.08–20.5)	14.6 (9.88–19.3)	14.8 (10.08–19.5)	0.58	0.89	0.88
AI%	12.1 (6.28–17.9)	10.3 (4.46–16.1)	11.7 (5.63–17.7)	22.20 (16.89–27.5)^b^	14.1 (8.79–19.4)b	16.4 (11.09–21.7)	0.01	0.32	0.24
P1, mmHg	105 (97.0–113)	101 (93.4–110)	101 (92.7–109)	108 (100.9–116)	107 (99.1–114)	108 (100.3–115)	0.53	0.34	0.21
P2, mmHg	110 (101.7–118)	105 (96.6–113)	105 (96.9–114)	116 (108.8–124)	112 (104.1–119)	113 (105.8–121)	0.23	0.15	0.21
ED, ms	272 (259–285)^b^ ^,^ ^c^	258 (245–271)	258 (244–271)	289 (277–301)^b^ ^,^ ^c^	266 (254–277)	268 (256–280)	0.06	0.24	0.37
Tr, ms	76 (62.4–89.6)^b^	60 (46.4–73.6)	63.8 (49.8–77.8)	105 (92.5–117.5)^b^ ^,^ ^c^ ^,^ ^d^	73.9 (61.4–86.4)	82.4 (69.9–94.9)	0.00	0.13	0.05
HR,bpm	78.5 (71.4–85.6)^b^	85.5 (78.7–92.9)	80.6 (73.3–87.9)	61.90 (55.3–68.5)^b^ ^,^ ^c^	75.70 (69.1–82.3)	71.70 (65.1–78.3)	0.00	0.04	0.07
aRPP,bpm xmm Hg	8529 (7512–9546)	8996 (7979–10013)	8476 (7441–9511)	7249 (6300–8199)^b^ ^,^ ^c^	8478 (7528–9427)	8166 (7217–9116)	0.07	0.45	0.65
RPP, bpm xmm Hg	9818 (8543–11093)	10429 (9153–11704)	9692 (8391–10993)	7929 (6741–9118)^b^ ^,^ ^c^	9575 (8386–10764)	9112 (7923–10300)	0.03	0.32	0.50
SEVR%	157 (142–172)	149 (135–164)	164 (149–180)	205 (191–218)^b^ ^,^ ^c^ ^,^ ^d^	176 (163–190)	190 (176–203)	0.00	0.02	0.01
Skin blood flow, PU	400 (202.7–597)	610 (412.5–807)	512 (306.9–718) #	288 (90.9–485)^b^ ^,^ ^c^ ^,^ ^d^	893 (695.5–1090)	614 (417–811)	0.42	0.05	0.47
cutaneous vascular resistance	0.27 (0.10–0.71)	0.25 (0.08–0.43)	0.21 (0.04–0.39)	0.55 (0.39–0.7)^b^ ^,^ ^c^	0.18 (−0.00–0.35)	0.26 (0.09–0.42)	0.02	0.53	0.70

Abbreviation: SBP, systolic blood pressure; DBP, diastolic blood pressure; PP, pulse pressure; AP, central augmented pressure; AI, unadjusted augmentation index; AI75, augmentation index adjusted to heart rate of 75 beats/minute; P1, first systolic pressure peak; P2, second systolic pressure peak; ED, ejection duration; Tr, reflection time; aRPP, central aortic rate–pressure product (heart rate × aortic systolic blood pressure); RPP, brachial rate–pressure product (heart rate × brachial systolic blood pressure); SEVR, subendocardial viability ratio.

^a^ The number listed for brachial SBP, under the “Cold Exposure” column corresponds to measurements taken 60 min after the cold exposure.

^b^ P < 0.05 baseline vs. exposure within that group.

^c^ P < 0.05 baseline vs. follow-up within that group.

^d^ P < 0.05 exposure vs. follow-up within that group.

# Missing information for skin blood flow in T2D: n = 1.

Our study also detected an attenuated increase (210 vs. 605 PU) in finger skin blood flow among persons with T2D in response to heat, indicative of less vasodilation (Stansberry et al., 1997). This finding was consistent with the observed lesser decrease in cutaneous vascular resistance in the heat. However, it should be noted that baseline levels of skin blood flow were lower in control compared to persons with T2D. These values were also less when compared to baseline conditions preceding cold exposure, which could indicate a difference in the placement of the probe affecting the readings (Kralj and Lenasi, 2022). Overall, a lesser increase in cutaneous blood flow in persons with T2D can be related to various factors associated with the diseases, such as endothelial dysfunction, increased vascular tone, microvascular changes, and peripheral neuropathy (Petrofsky, 2011; Tikhonova et al., 2022; Vinik et al., 1992).

In summary, the dampened cardiovascular responses observed in individuals with T2D during heat exposure in our study may be due to impaired autonomic regulation, compromised endothelial function, and heightened blood vessel stiffness, which can result in less pronounced vasodilation and a lesser increase in heart rate (Carrillo et al., 2016). These blunted cardiovascular responses could indicate a lesser capability to lose body heat and thus increase the risk of heat strain in warm environments.

### Strengths and limitations

The strength of our study lies in the fact that the participants were drawn from the general population, rather than from a more selected group, like a clinical sample. Additionally, we provided equal and strictly controlled exposures and measurements for all subjects. Furthermore, we controlled for the effects of certain confounders on arterial health, such as smoking, by selecting only non-smokers. This study has also some limitations. It is possible that seasonal acclimatization could have influenced the acute responses to cold and heat but estimate this impact to be minor. We acknowledge that T2D subjects had a higher BMI than controls, which could affect their thermal and cardiovascular responses to both cold and heat exposures. However, our sensitivity analyses did not support an influence of BMI on the obtained results. For safety reasons, participants were asked to continue normal use of their medications, including those for hypertension and diabetes. Therefore, we assessed the cardiovascular responses of individuals undergoing treatment for diabetes and hypertension, instead of studying these conditions without medical intervention. We acknowledge that the medication used for treating these conditions could also influence the observed cardiovascular responses. For example, calcium channel blockers were reported to be used by 50% of those with T2D and HTN and which could also partially explain the observed dampened cardiovascular responses observed in cold and heat. Furthermore, since many patients typically use multiple medications, it is difficult to attribute the observed cardiovascular responses to any single medication. However, this approach reflects the responses of the typical individuals with T2D and hypertension who is receiving treatment for these conditions. Additionally, individuals with T2D may have also been classified as having metabolic syndrome, but this was not evaluated in this study. It is also worth noting that assessing the effect of autonomic nervous system activity on cardiovascular responses could have provided additional understanding of the observed cardiovascular responses. We were also not unable to measure core temperature in our study, which could have provided additional insights of the degree of exposure and related cardiovascular responses. Furthermore, female participants were not included to our study, which restricts the generalizability of the results. We acknowledge that including post-menopausal females with T2D may have provided added information related to the association between environmental temperatures and cardiovascular responses. Finally, it should be noted that our results pertain to a specific population of individuals with T2D, and that those with a more severe progression of the disease may exhibit a greater state of cardiovascular dysfunction and vulnerability to temperature.

## Conclusion

Our study finds that persons with T2D have diminished cardiovascular responses both during short-term exposure to cold and heat. Consequently, having T2D may involve a higher risk of cold strain in low temperatures and heat strain in high temperatures. These responses could be even more substantial in those with marked vascular complications, metabolic dysfunction, and neuropathies, as well as individuals with T2D who are in occupations that involve prolonged exposure to extreme temperatures. Our findings are important because of the globally increasing number of individuals with T2D, together with the changing climate involving higher occurrence of weather extremes. The produced information can be useful for raising awareness among both patients with T2D and healthcare professionals about potential risks during extreme weather. Further research is needed to determine how T2D may heighten these risks, especially for women. Additionally, more research is warranted to elucidate the underlying neural, vascular, and metabolic mechanisms to address the cardiovascular challenges associated with thermal stress in this population.

## Data Availability

The raw data supporting the conclusions of this article will be made available by the authors, without undue reservation.
