# Supplementary material for: Blunted cardiovascular responses in individuals with type 2 diabetes and hypertension during cold and heat exposure
Source: Front Physiol. 2025 Apr 28;16:1558471. doi: 10.3389/fphys.2025.1558471 (PMC12066696; doi:10.3389/fphys.2025.1558471)
Supplement: Supplementary file 1 [file DataSheet1.docx]

Supplementary Material

Sensitivity analyses related to BMI and the cardiovascular responses related to cold and heat exposure

Table 1. Blood pressure heart rate and finger skin blood flow response in subjects with T2D and control in response to exposure to whole-body exposure to cold (10 °C) at rest, by considering BMI as covariate: The values represent Estimated Marginal Means (95% Confidence Interval) Calculated by Mixed-Effects models

|  | **T2D** | | | **Control** | | |
| --- | --- | --- | --- | --- | --- | --- |
| **Variable** | **Baseline**  N=10 | **Cold exposure**  N=8 | **Follow-up**  N=10 | **Baseline**  N=10 | **Cold exposure**  N=8 | **Follow-up**  N=9 |
| Central aortic SBP, mmHg | 114 (104-125)^a^ ^bc^ | 130 (119-141) * | 122 (112-133) | 115 (105126) ^abc^ | 155 (145-166) | 128 (118-139) |
| Central aortic DBP, mmHg | 78.7 (71.4-86) | 84.9 (77.3-92.5) | 80.2 (72.6-87.7) | 81 (73.70-88.3)^ac^ | 93.3 (85.8-100.9) | 79.7 (72.3-87.1) |
| Brachial SBP, mm Hg | 128 (116-139)^a^ | 142 (129-154)* | 133 (121-145) | 128 (116-140)^abc^ | 166 (154-178) | 138 (127-150) |
| Brachial DBP, mm Hg | 77.8 (72.8-85.4) | 83.8 (77.6-90.9) | 79 (73.5-86.9) | 79.9 (72.70-87.10)^ac^ | 92.7 (85.2-100.20) | 79 (71.70-86.3) |
| HR,bpm | 79.1 (72.5-85.6) a^b*^ | 74 (67.4-80.7) * | 69.6 (63-76.2) | 66.8 (60.3-73.3)^ab^ | 58.4 (51.7-65) | 60.2 (53.6-66.7) |
| aRPP,bpm xmm Hg | 8993 (8140-9847) c | 9429 (8547-10311) | 8428 (7551-9304) | 7744 (6891-8597) ^ac^ | 8999(8120-9877) | 7714 (6851-8577) |
| RPP, bpm xmm Hg | 10068 (9134-11002)* ^c^ | 10328 (9358-11297) | 9187 (8225-10149) | 8602 (7669-9535)^ac^ | 9641 (8676-10607) | 8368(7422-9314) |
| SEVR% | 156 (143-169)* | 158 (144-171)^b^* | 168 (155-182) | 193 (180-206) | 182 (168-195) | 183 (170-196) |

Abbreviation: SBP, systolic blood pressure; DBP, diastolic blood pressure; aRPP, central aortic rate–pressure product (heart rate × aortic systolic blood pressure); RPP, brachial rate–pressure product (heart rate × brachial systolic blood pressure); SEVR, subendocardial viability ratio.

^a^ P<0.05 baseline vs. exposure; ^b^ P<0.05 baseline vs. follow-up; ^c^ P<0.05 exposure vs. follow-up; * P<0.05 T2D vs. Control

Table 2. Blood pressure heart rate and finger skin blood flow response in subjects with T2D and control in response to exposure to whole-body exposure to heat (+40 °C) at rest, by considering BMI as covariate: The values represent Estimated Marginal Means (95% Confidence Interval) Calculated by Mixed-Effects models

|  | **T2D** | | | **Control** | | |
| --- | --- | --- | --- | --- | --- | --- |
| **Variable** | **Baseline**  N=10 | **Heat exposure**  N=8 | **Follow-up**  N=10 | **Baseline**  N=10 | **Heat exposure**  N=8 | **Follow-up**  N=9 |
| Central aortic SBP, mmHg | 110 (101.6-119)^a^ | 105 (96.6-114) | 107 (98.7-116) | 117 (109.1-124) ^a^ | 112 (104.4-120) | 114 (106.1-121) |
| Central aortic DBP, mmHg | 75.2 (67.7-82.7) | 72.8 (65.4-80.3) | 73.1 (65.6-80.7) | 82.3 (75.8-88.9)^a^ | 78.6 (72.1-85.2) | 75.2 (67.7-82.7) |
| Brachial SBP, mm Hg | 125 (113-137)^a^ | 121 (109-133)* | 121 (109-133) | 129 (119-139)^abc^ | 128 (117-138) | 128 (117-138) |
| Brachial DBP, mm Hg | 74.2 (66.8-81.7) | 71.5 (64-79) | 72 (64.5-79.6) | 81.5 (74.9-88)^ac^ | 77.2 (70.6-83.7) | 81.1 (74.5-87.6) |
| HR,bpm | 75.8 (68-83.5)* ^a^ | 83.4 (75.6-91.2) | 78.2 (70.3-86.2) | 63.3 (56.5-70.2)^ab^ | 77.1 (70.3-84) | 73.1 (66.3-80) |
| aRPP,bpm xmm Hg | 8274 (7120-9427) | 8773 (7620-9926) | 8397 (7231-9563) | 7450 (6437-8463) ^ab^ | 8679 (7666-9691) | 8367 (7354-9380) |
| RPP, bpm xmm Hg | 9408 (8033-10783) | 10077 (8702-11452) | 9465 (8070-10860) | 8257 (7048-9465) ^ab^ | 9903 (8694-11111) | 9439 (8231-10647) |
| SEVR% | 164 (150-179)* | 155 (141-170) | 170 (155-185) | 200 (187-212)^abc^ | 171 (159-184) | 185 (172-197) |

Abbreviation: SBP, systolic blood pressure; DBP, diastolic blood pressure; aRPP, central aortic rate–pressure product (heart rate × aortic systolic blood pressure); RPP, brachial rate–pressure product (heart rate × brachial systolic blood pressure); SEVR, subendocardial viability ratio.

^a^ P<0.05 baseline vs. exposure; ^b^ P<0.05 baseline vs. follow-up; ^c^ P<0.05 exposure vs. follow-up; * P<0.05 T2D vs. Control

**Thermal and cardiovascular responses to cold and heat among persons with T2D**


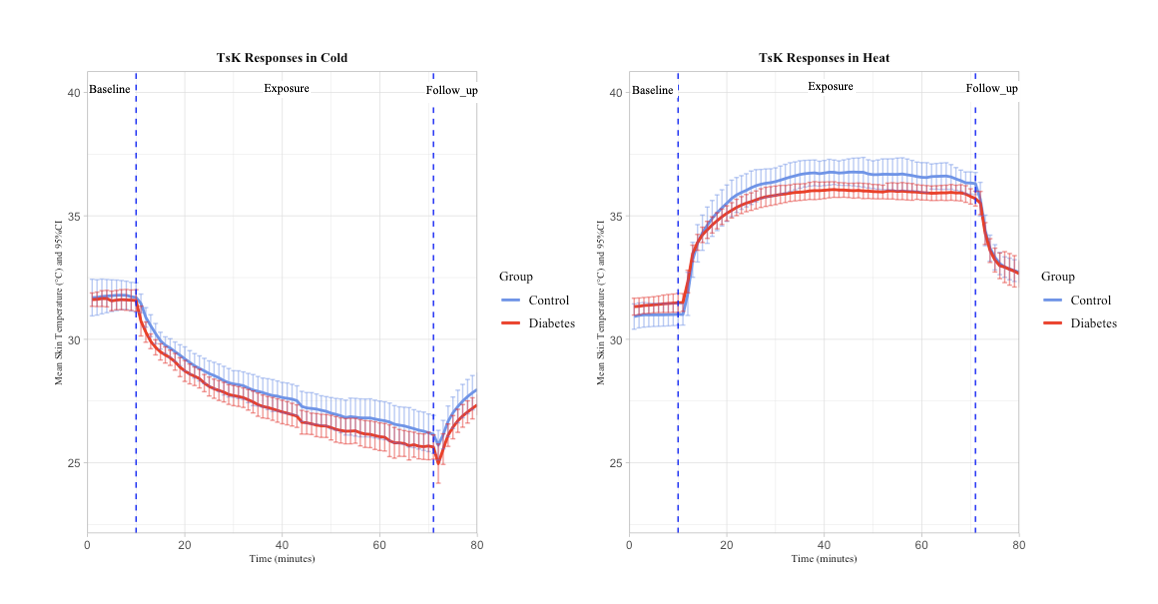


**Figure 1. Mean skin temperature (TsK) during whole-body exposure to cold and heat for each group, T2D and controls**


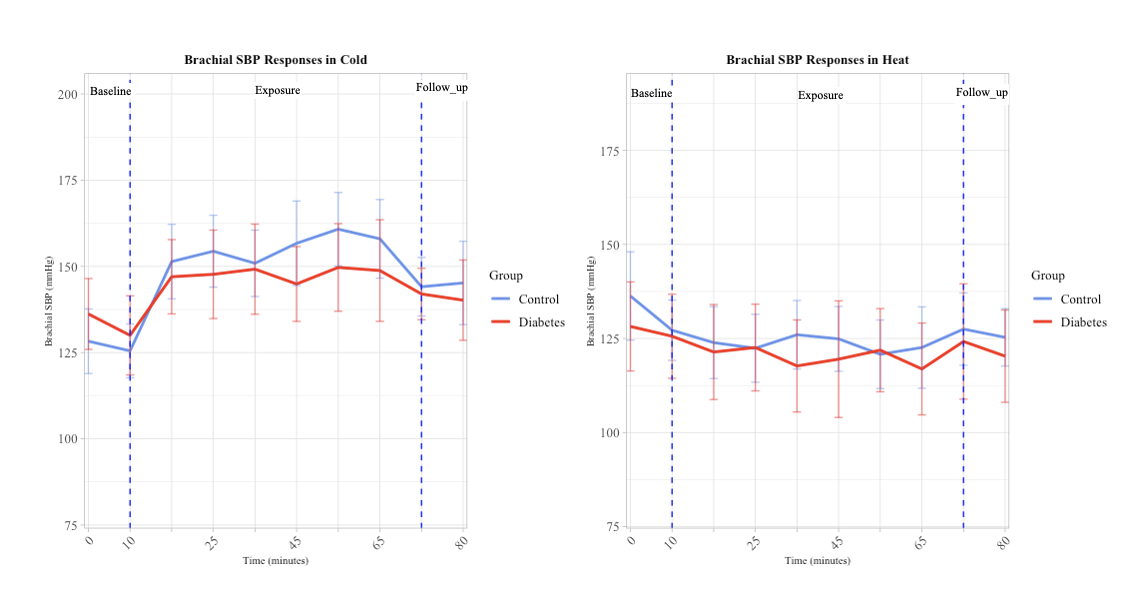


**Figure 2. Mean systolic blood pressure (SBP) during whole-body exposure to cold and heat for each group, T2D and controls**


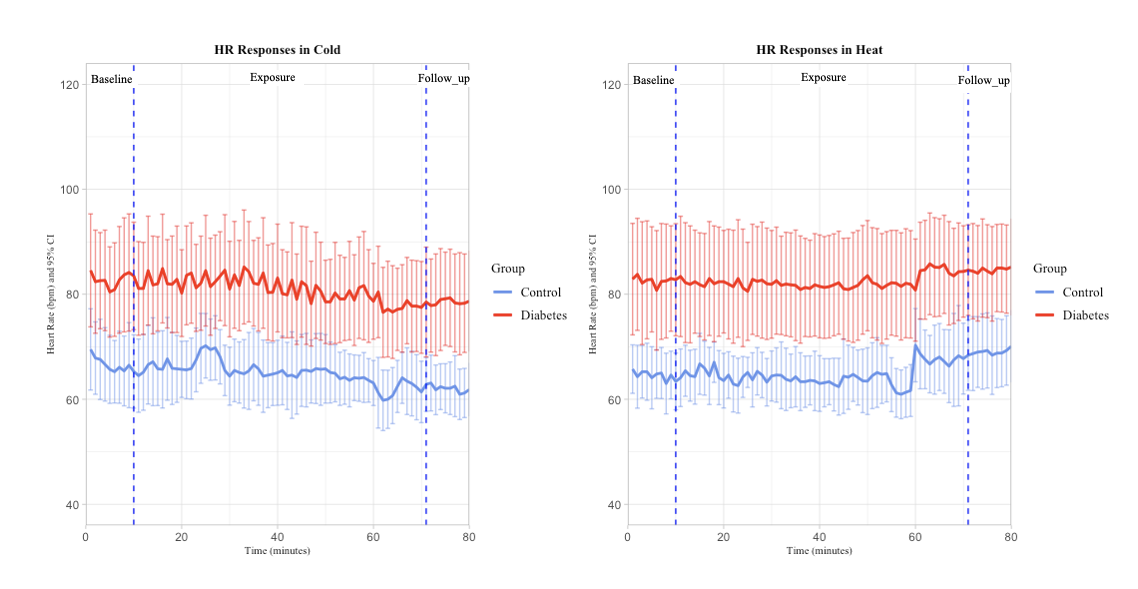


**Figure 3. Mean heart rate (HR) during whole-body exposure to cold and heat for each group, T2D and controls**


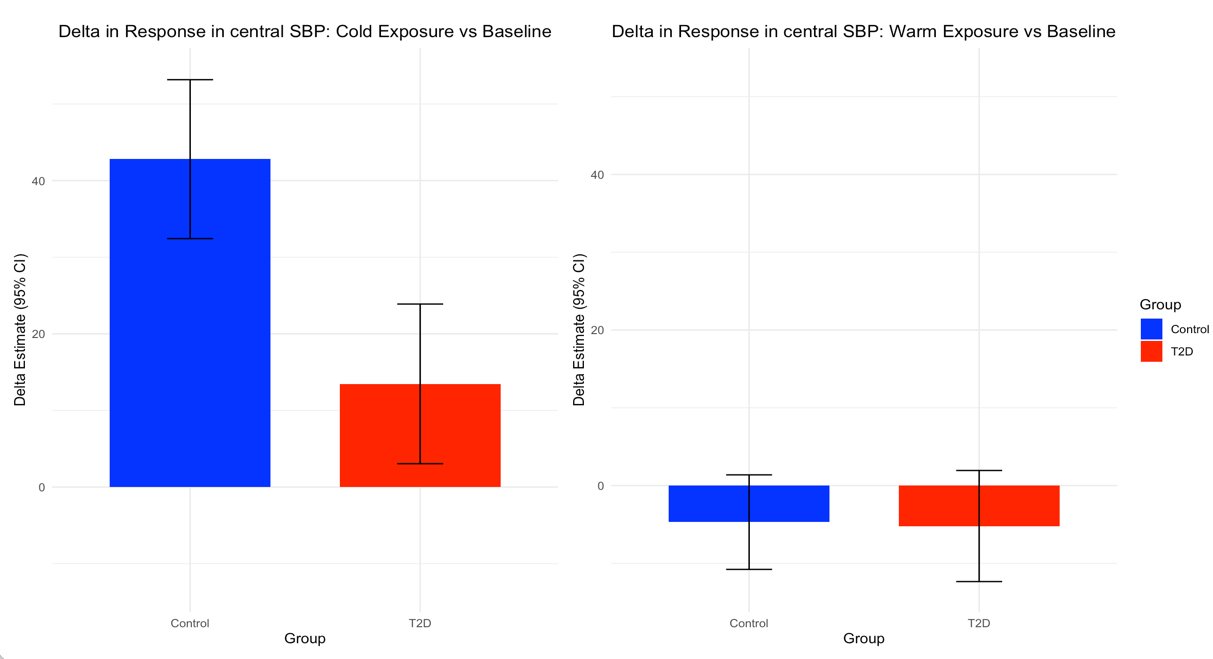


**Figure 4. Delta in responses from baseline to cold/heat exposure in central SBP (systolic blood pressure) for each group, T2D and controls**


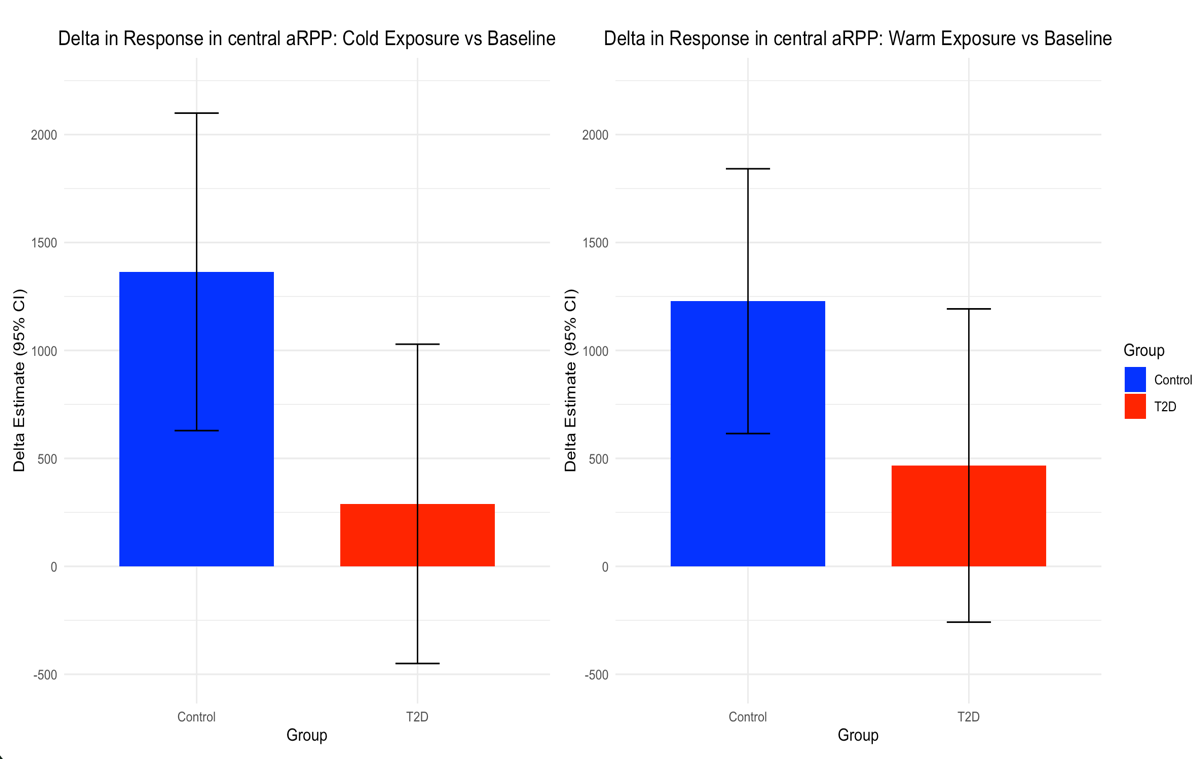


# Figure 5. Delta in responses from baseline to cold/heat exposure in aRPP (central aortic rate–pressure product) for each group, T2D and controls


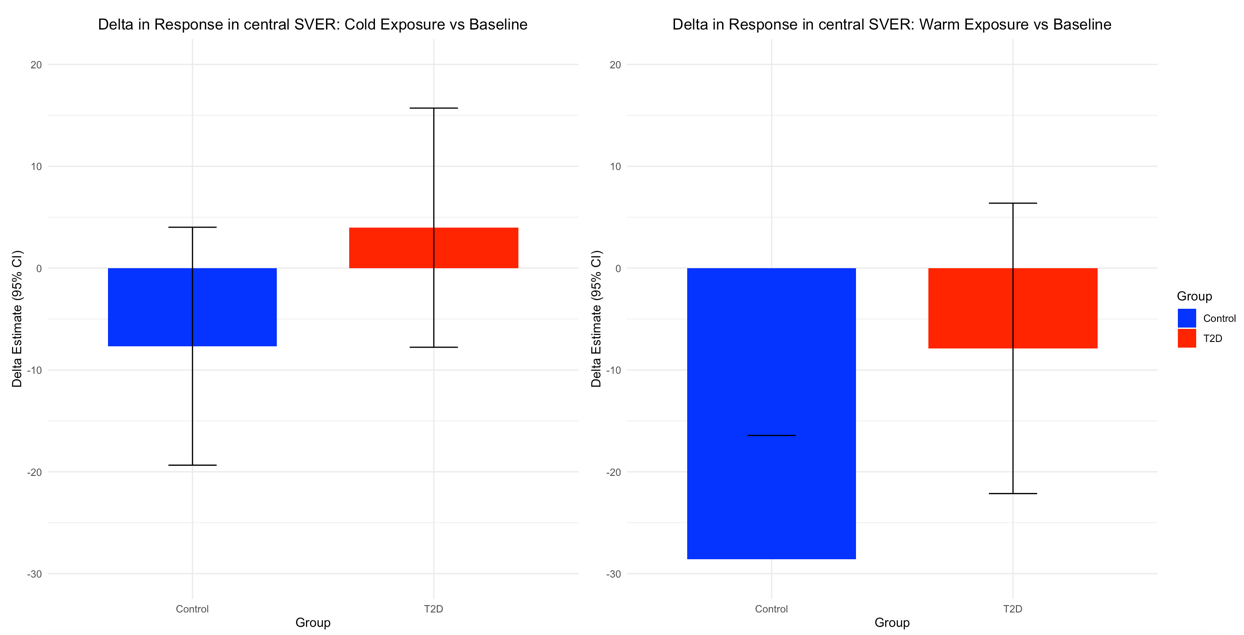


# Figure 6. Delta in responses from baseline to cold/heat exposure in SEVR (subendocardial viability ratio) for each group, T2D and controls


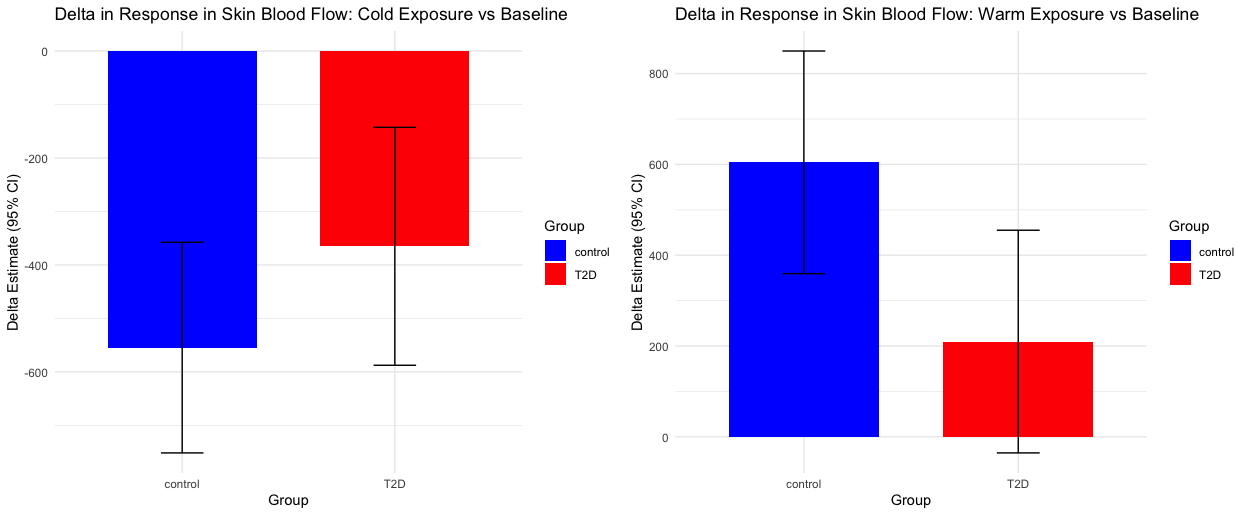


# Figure 7. Delta in responses from baseline to cold/heat exposure in Skin Blood Flow for each group, T2D and controls


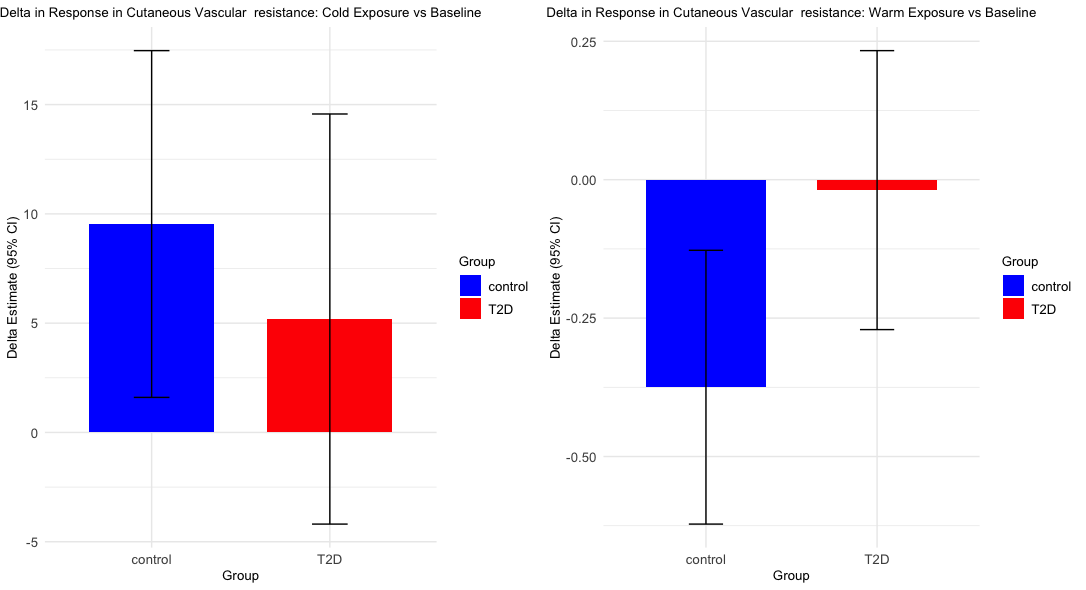


# Figure 8. Delta in responses from baseline to cold/heat exposure in Cutaneous Vascular resistance for each group, T2D and controls
